# Extraosseous Intradural Chondrosarcoma of the Cervical Spine: A Case Report with Brief Review of Literature

**DOI:** 10.1155/2018/6921020

**Published:** 2018-02-28

**Authors:** Elizabeth Presutto, Sejal Patel, Joseph Fullmer, Sajeev Ezhapilli

**Affiliations:** ^1^Department of Radiology, SUNY Upstate Medical University, Room 3530, 750 East Adams Street, Syracuse, NY 13210, USA; ^2^Department of Pathology, SUNY Upstate Medical University, 750 East Adams Street, Syracuse, NY, USA

## Abstract

Mesenchymal chondrosarcoma (MCS) is a malignant cancer of the cartilage that accounts for less than 1% of all chondrosarcomas and typically occurs within the bone. One-third of all mesenchymal chondrosarcomas are extraosseous soft tissue sarcomas, rendering this as an uncommon entity. We report a rare case of an extraosseous chondrosarcoma with the cervical spinal canal in a 21-year-old male. The purpose of this case report is to discuss the imaging characteristics of this pathology proven diagnosis.

## 1. Introduction

Extraosseous mesenchymal chondrosarcoma (MCS) is an aggressive, high-grade malignant tumor predominantly of the bone and also of the soft tissue that demonstrates a biphasic pattern of small cells with islands of atypical cartilage. It represents 1% or less of all chondrosarcomas, in which one-third are extraosseous [1]. Most cases of MCS originate in the head and neck region, followed by trunk and extremities. Craniofacial, spine, and ribs are most commonly affected [2]. MCS affects all ages, with a peak incidence in the second decade of life and a slight predilection for women. Overall survival rate is poor with a 10-year survival of less than 30% [3, 4].

Extramedullary, intradural location of MCS is exceedingly rare with a limited number of cases reported in the United States [5–7]. Herein, we report a case of extraosseous mesenchymal chondrosarcoma in a young male involving the cervical spinal canal.

## 2. Case Presentation

**Figure 1:**
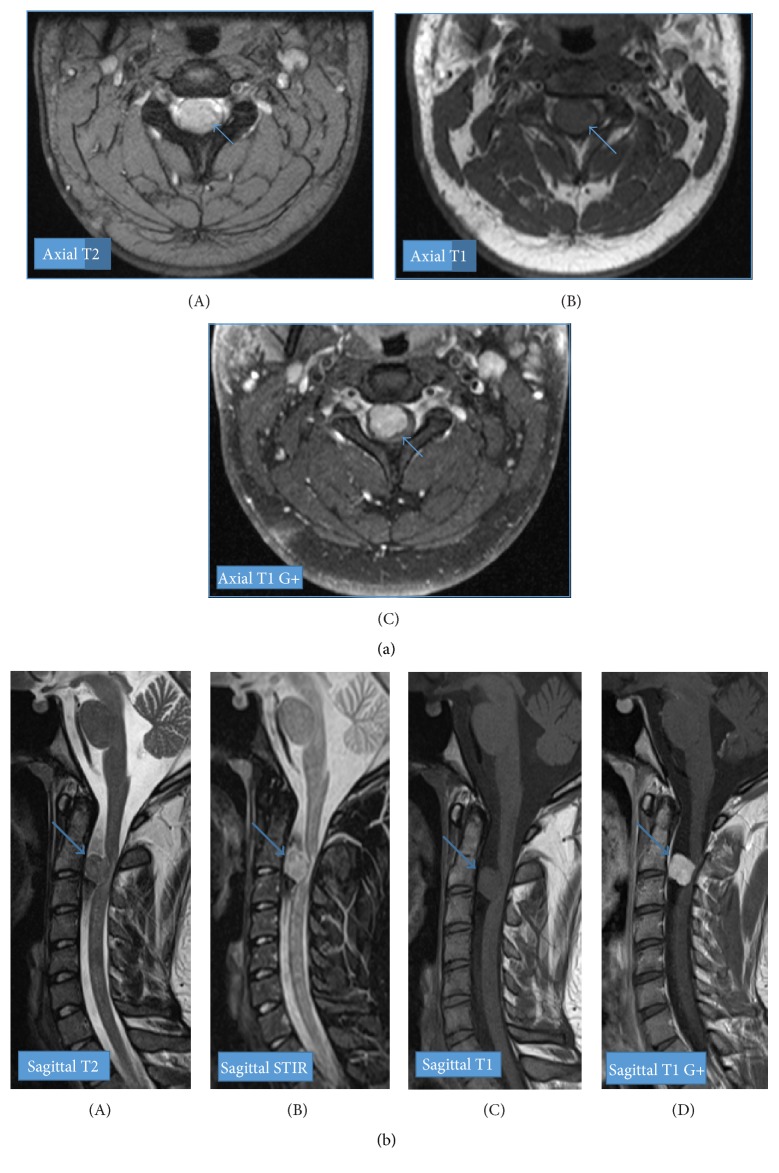
There is a slightly lobulated well-demarcated 1.4 × 1.7 × 1.2 cm intradural extramedullary mass lesion (blue arrow) in the spinal canal, anterior to the spinal cord at the level of C2-C3. There is mass effect with posterior displacement of the cord as well as cord edema. There is widening of the ventral subarachnoid space. It is homogeneously isointense on T1 and slightly heterogeneously hypointense on T2. Postcontrast imaging demonstrates avid diffusely homogenous enhancement.

A 21-year-old white male presented to his primary care provider with a 3-month history of neck pain with stiffness, tingling, and paresthesias in his right arm at the C6–C8 dermatomal distribution. He also complained of loss of dexterity, loss of grip strength, and some gait instabilities. His exam was positive for Babinski's sign on the right and some vision loss. An initial brain magnetic resonance imaging (MRI) was normal. Contrast enhanced cervical spine MRI demonstrated an enhancing intradural extramedullary T1 isointense and T2 hypointense mass at the level of C2-C3, anterior to cervical spinal cord and resulting in severe spinal canal stenosis. There is mass effect with posterior displacement of the cord, cord compression, and cord edema (Figures 1(a) and 1(b)).

**Figure 2:**
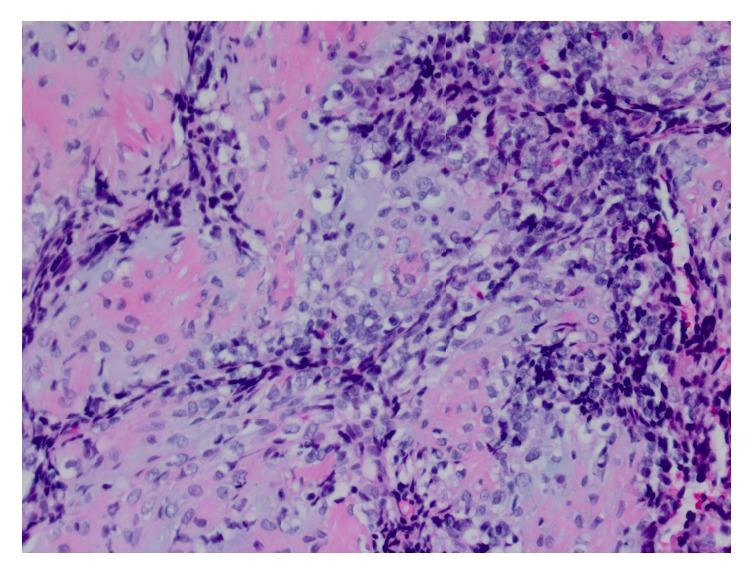
10x magnification of chondroid and mesenchymal portions.

**Figure 3:**
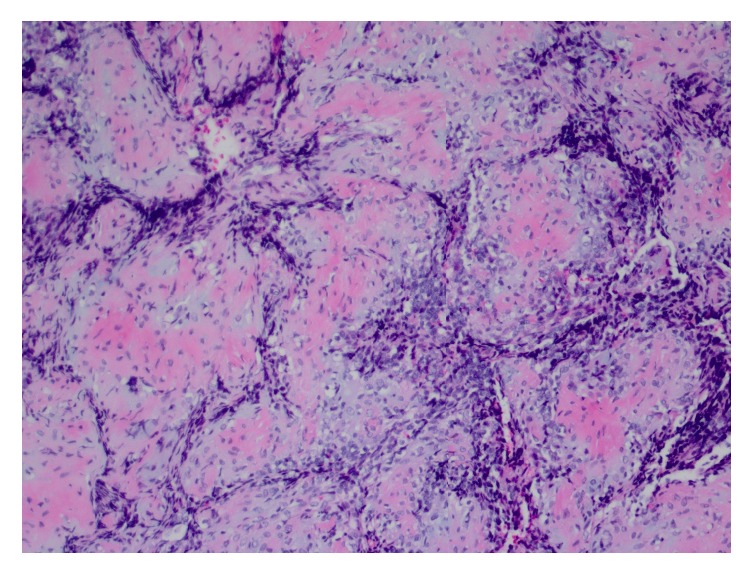
10x magnification of chondroid portion.

**Figure 4:**
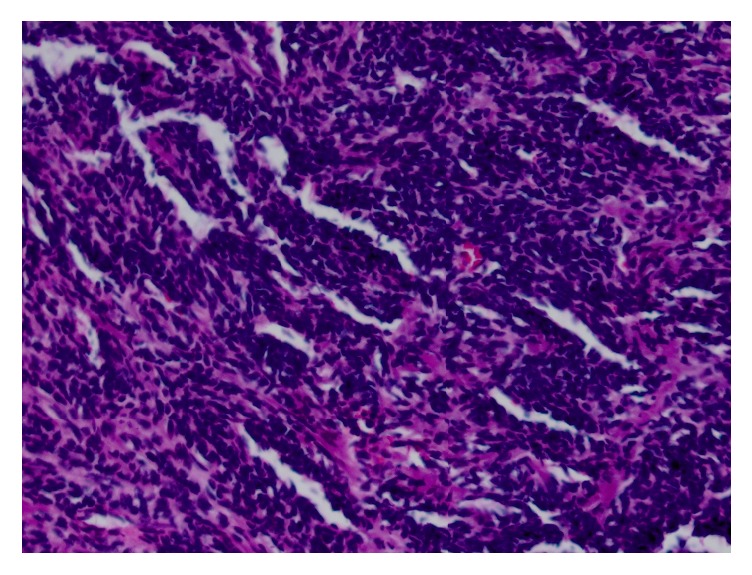
20x magnification of mesenchymal portion.

Subsequently, decompression with bilateral posterior cervical C2-C3 laminectomies and tumor resection was attempted by neurosurgery. However, full resection of the mass was not achieved after significant loss of motor signals was evoked. As a result, the patient only received posterior decompression at this level. The patient's postoperative course was uneventful and the patient was discharged with plan to attend occupational and physical rehabilitation. On pathology, gross section demonstrated fragments of tissue ranging from 0.1 to 0.4 cm. Microscopy revealed tightly packed small blue cells with greatly increased nuclear to cytoplasmic ratios and some areas of cartilage. Pathology was sent to an outside institution for further evaluation. It was determined to show biphasic pattern with densely cellular regions of small anaplastic cells and other areas of chondroid differentiation, consistent with diagnosis of mesenchymal chondrosarcoma (Figures 2–4).

**Figure 5:**
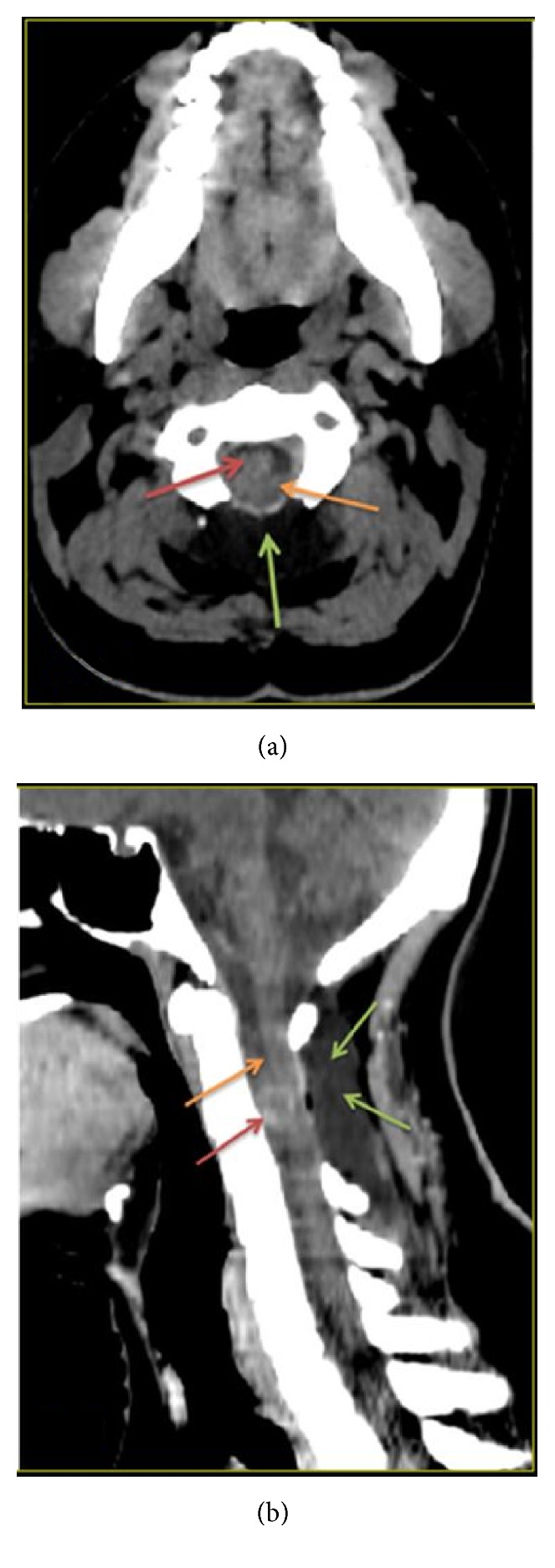
Axial NECT (Figure 5(a)) and sagittal NECT (Figure 5(b)) of cervical spine demonstrate surgical changes secondary to posterior spinal decompression (green arrows) at C2 and C3 levels, as well as a well-defined soft tissue density lesion (red arrows) anterior to cervical spinal cord (orange arrows) at C2-C3.

The patient subsequently underwent definitive radiotherapy followed by adjuvant chemotherapy with an Ewing sarcoma protocol for a total of one year. Nonenhanced CT (NECT) of cervical spine was performed at an outside institution for radiotherapy planning (Figures 5(a) and 5(b)). Most current follow-up at two years demonstrates stable findings on MRI with no new abnormalities. The patient has persistent weakness in the left arm but denies any new neurologic symptoms.

## 3. Discussion

Mesenchymal chondrosarcoma (MCS) is a rare malignant tumor that is classified as chondrosarcoma due to its focal appearance of cartilage with alternating undifferentiated stroma. The first two examples of MCS were described by Lichtenstein and Bernstein in 1959 [8]. Mesenchymal chondrosarcoma represents a rare subset of chondrosarcoma, only few of which are extraskeletal in nature [9]. Shapeero et al. reported that only 7 of 224 cases of MCS were extraosseous in origin [10]. It tends to grow rapidly, predominantly affects teens and young adults, and usually involves bone and somatic soft tissue [4].

**Figure 6:**
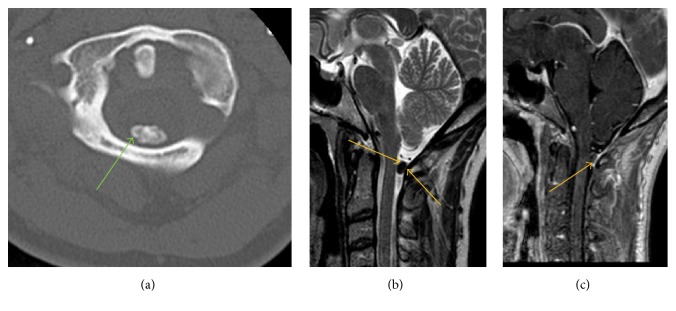
Axial NECT of cervical spine (Figure 6(a)) demonstrates a calcified well-marginated lesion (green arrow) within posterior aspect of central canal at C1 level. On sagittal T2WI (Figure 6(b)) and sagittal postgadolinium (Figure 6(c)) MR images, this extramedullary intradural lesion (orange arrows) is noted to be T2 hypointense and mildly rim enhancing, respectively, causing effacement of posterior thecal sac and abutment of cervical spinal cord at the level of posterior arch of C1. The imaging findings favor a calcified meningioma.

**Figure 7:**
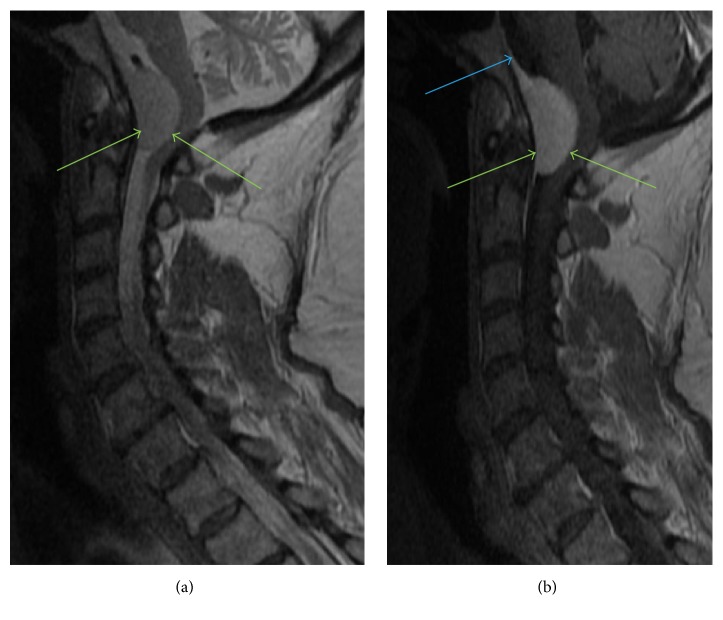
Sagittal T2WI (Figure 7(a)) and sagittal postgadolinium images (Figure 7(b)) show a homogeneously enhancing T2 hyperintense dural-based lesion (green arrows) representing a meningioma, occupying the anterior aspect of foramen magnum. This lesion demonstrates a characteristic dural tail (blue arrow) on postgadolinium image. This lesion causes effacement of anterior subarachnoid space and exerts mass effect resulting in posterior displacement of the adjacent medulla and upper cervical spinal cord with impingement at the level of foramen magnum.

**Figure 8:**
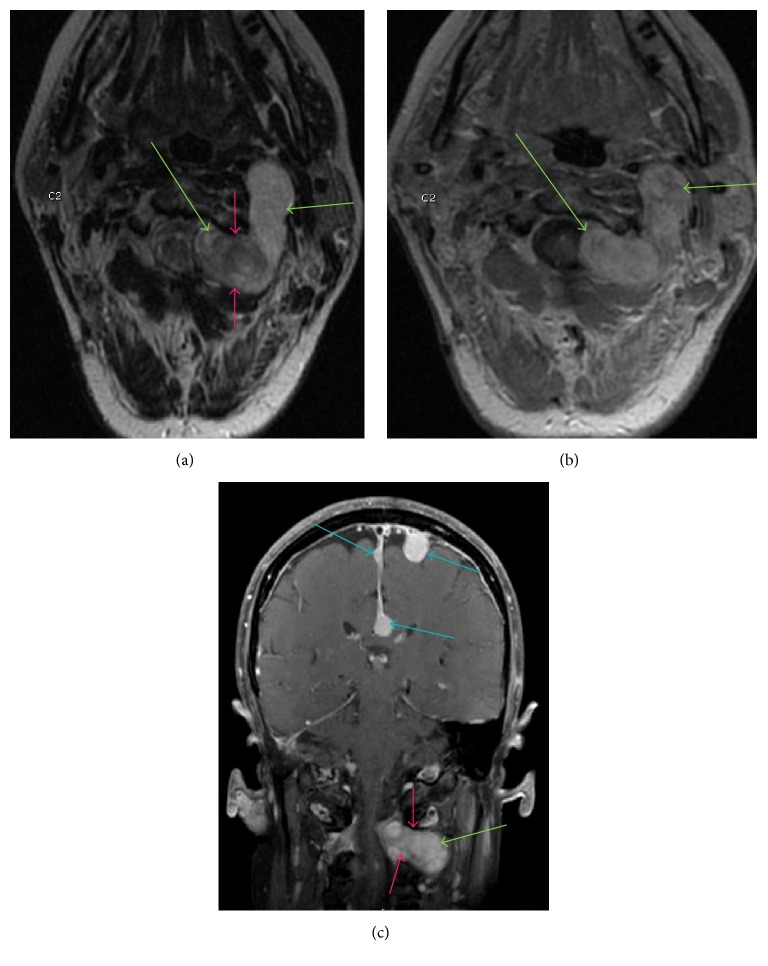
Axial T2WI (Figure 8(a)), axial postgadolinium (Figure 8(b)), and coronal postgadolinium (Figure 8(c)) demonstrate a well-defined extramedullary heterogeneously enhancing T2 hyperintense lobulated lesion representing a schwannoma (green arrow) extending along the left C2 nerve root in this patient with neurofibromatosis type 2 (NF2). This lesion causes effacement of left lateral thecal space at C1-C2 level and abuts the cervical spinal cord on the left, which is demonstrated on Figures 8(a) and 8(b). There is widening of left C1-C2 neural foramen (pink arrow) by this lesion. Additional multiple intracranial meningioma (blue arrows) are also identified on coronal postgadolinium image (Figure 8(c)) in this patient with NF2.

Given the extraosseous presentation of our case, MCS was not initially suggested. Extraskeletal MCS is exceedingly rare, representing about 0.33% of all chondrosarcomas [2]. Chondrosarcomas generally occur in the pelvis, shoulder, or proximal femur and present in the 4th or 5th decade of life [11]. Despite the mesenchymal type often in the head and neck region, the rarity of the disease and unlikeliness to occur in the soft tissue resulted in its nonconsideration made for initial differential diagnosis. Initial preoperative imaging differential diagnoses instead included meningioma and schwannoma. Meningioma typically presents on MRI as well-defined, extra-axial dural-based mass that is isointense to hypointense to gray matter on T1 demonstrating avid, homogenous contrast enhancement after gadolinium administration, similar to the imaging findings in our case (Figures 6 and 7) [12]. Given the symptoms of sensory loss and weakness, a peripheral nerve tumor such as schwannoma was also considered [12]. Classic enhancement patterns on MRI have been described for extracranial schwannomas. Characteristically, schwannomas present with findings such as the target sign, fascicular appearance, and hypointense rim on T2-weighted images (Figure 8) [13, 14]. Furthermore, vestibular schwannomas may be differentiated from a cerebellopontine angle meningioma by the predilection of schwannomas to demonstrate microhemorrhages on T2-weighted imaging [15].

MRI is the preferred imaging modality for intraspinal tumors, but there is no pathognomonic description for extraosseous MCS. Chondrosarcomas share similar findings with chordomas, making the differential diagnosis challenging [16]. On imaging, extraosseous MCS can present as calcified area of low intensity on both T1-weighted and T2-weighted MRI images, whereas the noncalcified areas present as low and high intensity on T1-weighted (T1WIs) and T2-weighted images (T2WIs), respectively. Therefore, the two components typically show clear demarcation on T2WIs and can be used in differentiation. However, Bae et al. reported a case of intraspinal MCS with no evidence of calcification, which elucidates the variation and difficulty in diagnosis by imaging. They described findings of isointensity to the spinal cord on T1WIs and high intensity or isointensity on T2WIs [16]. An additional study of imaging in 10 extraskeletal MSC patients found that, on gadolinium-enhanced MRI, MCS is expected to show heterogeneous enhancement in both calcified and uncalcified areas, although sensitivity and specificity of this finding are unknown [17]. Harsh IV and Wilson reviewed 16 MCS in the primary central nervous system, of which 5 occurred indurally. These tumors typically occur at an extramedullary, extradural site, but majority occur with dural attachment making this case of extramedullary, intradural MCS even more rare [18].

## 4. Management

Due to the rarity of the disease, no universal management protocol has been established. Total resection with wide margins is typically suggested and surgery has shown to be essential for a positive outcome. Adjunctive chemotherapy and/or radiotherapy may be considered; however, little evidence for their utility has been demonstrated so far. Careful, long-term follow-up and monitoring should last at least 10 years due to the tendency toward recurrence and metastasis in >70% of cases [9, 18].

## 5. Conclusion

Our case demonstrated an unusual and rare extraskeletal location of mesenchymal chondrosarcoma: extramedullary, intradural within the cervical spine. We suggest the importance of considering the diagnosis of mesenchymal chondrosarcoma when aforementioned diagnostic imaging features are witnessed.
